# Does colon cancer ever metastasize to bone first? a temporal analysis of colorectal cancer progression

**DOI:** 10.1186/1471-2407-9-274

**Published:** 2009-08-07

**Authors:** Eira S Roth, David T Fetzer, Bruce J Barron, Usha A Joseph, Isis W Gayed, David Q Wan

**Affiliations:** 1 Medical Students, The University of Texas Medical School, Houston, USA; 2 Visiting Professor, University of Texas Medical School, Houston, USA: Emory University hospital Midtown, 550 peachtree street, NE Atlanta Georgia 30308, USA; 3 Department of Diagnostic and Interventional Imaging, The University of Texas Medical School, Houston, MSB 2.130B, 6431 Fannin Street, Houston, Texas 77030, USA

## Abstract

**Background:**

It is well recognized that colorectal cancer does not frequently metastasize to bone. The aim of this retrospective study was to establish whether colorectal cancer ever bypasses other organs and metastasizes directly to bone and whether the presence of lung lesions is superior to liver as a better predictor of the likelihood and timing of bone metastasis.

**Methods:**

We performed a retrospective analysis on patients with a clinical diagnosis of colon cancer referred for staging using whole-body ^18^F-FDG PET and CT or PET/CT. We combined PET and CT reports from 252 individuals with information concerning patient history, other imaging modalities, and treatments to analyze disease progression.

**Results:**

No patient had isolated osseous metastasis at the time of diagnosis, and none developed isolated bone metastasis without other organ involvement during our survey period. It took significantly longer for colorectal cancer patients to develop metastasis to the lungs (23.3 months) or to bone (21.2 months) than to the liver (9.8 months). Conclusion: Metastasis only to bone without other organ involvement in colorectal cancer patients is extremely rare, perhaps more rare than we previously thought. Our findings suggest that resistant metastasis to the lungs predicts potential disease progression to bone in the colorectal cancer population better than liver metastasis does.

## Background

Colorectal cancer remains the third most common cancer among adult men and women in the United States and the third most common cause of death from cancer [1,2]. It is well accepted that colorectal cancers metastasize to the liver and lungs more frequently than to bone or other organs [2]. This pattern of involvement has been attributed both to the pattern of blood flow from the colon to the portal system and to molecular signal proteins inherent to the microcosm of each organ system; however; whether one component is more important than the other is still under debate [2-4]. Many studies have focused on characterizing which organ systems are most affected by colorectal cancer metastasis; however, it remains unclear whether there is a specific temporal pattern for metastasis.

If isolated bone metastasis is truly rare in colorectal cancer patients, then clinicians can be very conservative in evaluating a suspected bone lesion without other organ involvement. Because bone metastasis often indicates the terminal phase of colon cancer, clinicians should be more vigilant about possible bone metastasis in colorectal cancer patients with lung metastasis.

The aim of this retrospective study was to establish whether colorectal cancer can bypass other organs and metastasize directly to bone and whether lung metastasis is better than liver metastasis for predicting whether and when bone involvement develops. In addition to determining which other metastasis more effectively predicts impending bone lesions, we determine whether the presence of liver or lung lesions correlates with the increased likelihood and the timing of bone metastasis.

## Methods

We submitted an application to The University of Texas Medical School at Houston Institutional Review Board, which granted us permission to access patient records for this study from 3 outlying medical facilities. We acquired information retrospectively and gave patient a random numerical code for identification purposes.

We searched the serial imaging reports of all patients referred to these outlying medical facilities from January 2000 to December 2008 with F-18-flurodeoxyglucose (FDG)-PET in correlation with a recent CT or PET/CT and found those with a clinical diagnosis of colon cancer. We analyzed a total of 703 reports from colon cancer patients (an average of 3.04 reports per patient). We attempted to have PET and CT scans for all patients with a diagnosis of colon cancer for staging/restaging or treatment monitoring. Some colon cancer patients never had PET/CT scans because of variable clinical issues or insurance status and these patients were not included in this study. Stage of the patients ranged from stage I to stage IV. We excluded patients if they had a second primary cancer. Thus, we included 252 patients with a primary diagnosis of colorectal cancer in this retrospective study. This group contained 122 female patients (48.4%) and 130 male patients (51.6%).

We included all 252 individuals when we determined organ involvement. However, when calculating the time and sequence of metastatic spread, we found that 21 of the 252 patients had presented solely for initial staging and excluded them from that analysis. The 231 individuals had a known date of initial diagnosis and the time of new metastasis noted in the serial imaging reports during the investigation. Of the 102 patients with organ metastasis, 71 patients received further chemotherapy; 12 patients received combined chemotherapy and surgery; 16 patients received combined chemotherapy and radiation therapy; 2 patients received surgery only; and 5 patients received no therapy. The status of treatment for 5 patients after metastasis was uncertain. Nineteen patients developed new organ metastasis only at the end of our survey periods. Time to development of metastatic disease was defined as time from initial diagnosis to the appearance of metastasis in imaging studies.

To minimize the impact of institutional variability, we included imaging studies only if they had been read originally by 1 of 3 certified nuclear medicine radiologists.

### Image Interpretation

Board-certified nuclear medicine radiologists initially interpreted the imaging studies by using a GE Advanced Workstation for PET/CT cases or MIM vista fusion program (MIM vista Corp) to analyze PET and CT images before we conceived and conducted this study. Another individual subsequently extracted information from the reports to include in this study. Metastases to the liver, lungs, and bone were recorded. Because PET has low sensitivity for detecting brain tumors, we did not assess brain metastases. Disease progression was documented when an imaging report showed involvement of a new organ system. If a metastasis resolved after the patient received therapy, the metastasis was still recorded based on the date on which it was first identified by imaging.

One certified nuclear medicine radiologist reevaluated any inconclusive or contradictory information and confirmed the findings if lesions were visible on subsequent imaging studies or if other imaging modalities confirmed the presence of the lesions. We excluded imaging studies if we could not find a subsequent imaging report to confirm the lesion.

### Statistics

Data were expressed as mean ± SD. We used the Microsoft Excel 2003 to calculate the confidence intervals. We determined the statistical significance of differences by using an unpaired 2-tailed Student *t *test and considered *P *values of less than 0.05 to be statistically significant. Because we focused on the temporal pattern of the onset time of organ metastasis, we did not perform Kaplan-Meier survival analysis for this study.

## Results

Of the 252 patients, 55 (22%) received adjuvant local radiation therapy and 195 (77%) underwent adjuvant chemotherapy in addition to the surgical removal of the primary tumor. The mean patient age was 64.2 years at the time of initial diagnosis, with a standard deviation of 11 years. The average follow-up period after diagnosis was 38 months, and 21 of 252 patients (8.3%) presented solely for initial staging. Table 1 shows stages and therapy.

**Table 1:** Demographic information of the survey population

*Parameter*	*Number (percentage)*
Age (mean ± SD)	64.2 year ± 11
Initially staged as I to III	216/252 (86)
Initially staged as IV	36/252 (14)
Primary cancer surgery	206/231 (89)
Adjuvant radiation therapy	55/231 (23.8)^1^
Adjuvant chemotherapy	195/231 (84.4)^1^
Total metastasis to liver, lung, or bone	102/252 (40)

SD = Standard deviation

^1 ^The denominator is the 21 patients seen only for initial staging subtracted from the total number of patients.

Organ involvement in the 252 patients comprised metastases to the liver, lung, and bone. There was little difference between male and female patients (data not shown). Overall, 102 of the 252 patients (40%) had organ metastasis.

Sixty-three of the 252 subjects (25%) developed lung metastasis, and 75 of 252 individuals (30%) developed liver metastasis. Only 4 of 63 patients (6.3%) with lung metastases had them at diagnosis, whereas 21 of the 75 (28%) with liver metastasis did (Table 2). Twenty-three of the 63 lung metastasis patients (36.5%) developed lung metastasis first without other organ involvement, whereas 48 of 75 patients (64%) had colorectal cancer that metastasized to the liver without first involving other organs. Forty-nine of the 63 lung metastasis patients (77.8%) had concurrent other metastases, whereas 42 of the 75 liver metastasis patients (56%) did. Fourteen of the 63 subjects (22%) with lung metastasis remained free of metastasis to other organs during follow-up, whereas 33 of the 75 patients (44%) with liver metastasis did. Only 2 individuals developed lung metastasis before subsequent liver metastasis, whereas others showed lung infiltration more slowly, years after initial diagnosis.

**Table 2:** Colorectal cancer organ metastases in certain criteria

	*Patient Number (Percentage)*			
	
*Organ Metastasis*	*Total metastasis*	*At initial diagnosis*	*Concurrent with other metastasis*	*As the first site of organ metastasis*
Liver	75/252 (30)	21/75 (28)	42/75 (56)	48/75 (64)
Lung	63/252 (25)	4/63 (6.3)	49/63 (77.8)	23/63 (36.5)
Bone	14/252 (5.5)	1/14 (7.1)	14/14 (100)	0/14 (0)

Analysis of metastasis to bone showed 14 of the 252 individuals (5.5%) had bone lesions and no individuals had metastasis only to bone at the time of diagnosis. No patient developed bone metastasis without liver and/or lung metastasis. One individual presented with bone lesions at the time of diagnosis; however, liver metastasis was also present.

Of the 14 patients with lesions to bone, 8 (57%) had both liver and bone involvement, whereas 10 (71%) had lung metastasis. Fifty-three of the 63 patients (84%) with lung lesions remained free of metastasis to bone during follow-up. Similarly, 63 of the 75 patients (84%) with liver metastasis also remained free of metastasis to bone during follow-up.

The average time from initial diagnosis to detection of metastatic disease was 9.8 months (± 14.9, confidence interval [CI] 6.2–13.3) for liver involvement, significantly shorter than the average times of 23.3 months (± 25.3, CI 16.4–30.1) to detect lung metastasis and 21.2 months (± 18.5, CI 11.2–31.3) to detect metastasis to bone (Table 3), *P *< 0.05. The average time for colon cancer to metastasize from the liver to bone was 8.3 months (± 13.4, CI 0.6–15.9), whereas the time to metastasize from lung to bone was 3.3 months (± 4.2, CI 0.7–5.9). However, this difference is not significant because of the small sample size. Among the 8 patients who exhibited metastases to liver, lungs, and bone, the average time for the cancer to metastasize from the liver to bone was also longer than the time for the cancer to metastasize from the lung to bone (6.0 months ± 11.1 versus 4.3 months ± 6.1). However, the results are not statistically significant. On average, involvement was detected in all 3 organs within 30 months of the original diagnosis of colon cancer.

**Table 3:** Average time (months) to organ metastasis in metastatic colon cancer patients

*Type of Metastasis*	*Time ± SD*	*Patients*^1^
Liver metastasis	9.8 ± 14.9	54
Lung metastasis	23.3 ± 25.3^2^	59
Bone metastasis	21.2 ± 18.5^2^	13
From liver metastasis to bone metastasis	8.3 ± 13.4	12
From lung metastasis to bone metastasis	3.3 ± 4.2^3^	10

SD = standard deviation.

^1 ^Patients with only 1 scan are not included in these calculations.

^2 ^A significant difference (unpaired Student *t *test, *P *< 0.05) was found when the time to metastasis from the colon to the lung or bone was compared with the time to metastasis from the colon to the liver.

^3 ^No significant difference (*P *> 0.05) was found when the time from lung metastasis to bone metastasis was compared with the time from liver metastasis to bone metastasis.

Of the 14 patients with metastasis to bone, 2 were still alive, 2 were lost to follow-up, and 8 were dead by the end of this study. The average time from when bone involvement was detected until the patient died was 15.9 months (± 13.2, CI 6.7–25.1), and the average lifespan of patients with bone metastasis after initial diagnosis of colorectal cancer was 42.4 months (± 18.1, CI 29.8–54.9).

## Discussion

### Analysis of Involvement in Bone

This study determined that despite individual variance in the degree and order of organ involvement among patients with colorectal cancer, a general temporal pattern does exist. Malignancies never spread primarily to bone. This is a characteristic particular to colorectal cancer as bone metastasis is far more frequent in the other leading cancer types. Although clinicians still cannot agree on the prevalence of breast, lung, and prostate cancers metastasis only to bone is a common occurrence in these cancers [5]. One study even postulated that as many as 70% of patients with stage IV breast cancer have bone involvement [6]. By contrast, cancer typically metastasizes to bone in less than 10% of colorectal cancer patients [7]. This suggests that colon cancer behaves differently with respect to bone metastasis compared with other cancers.

**Figure 1:**
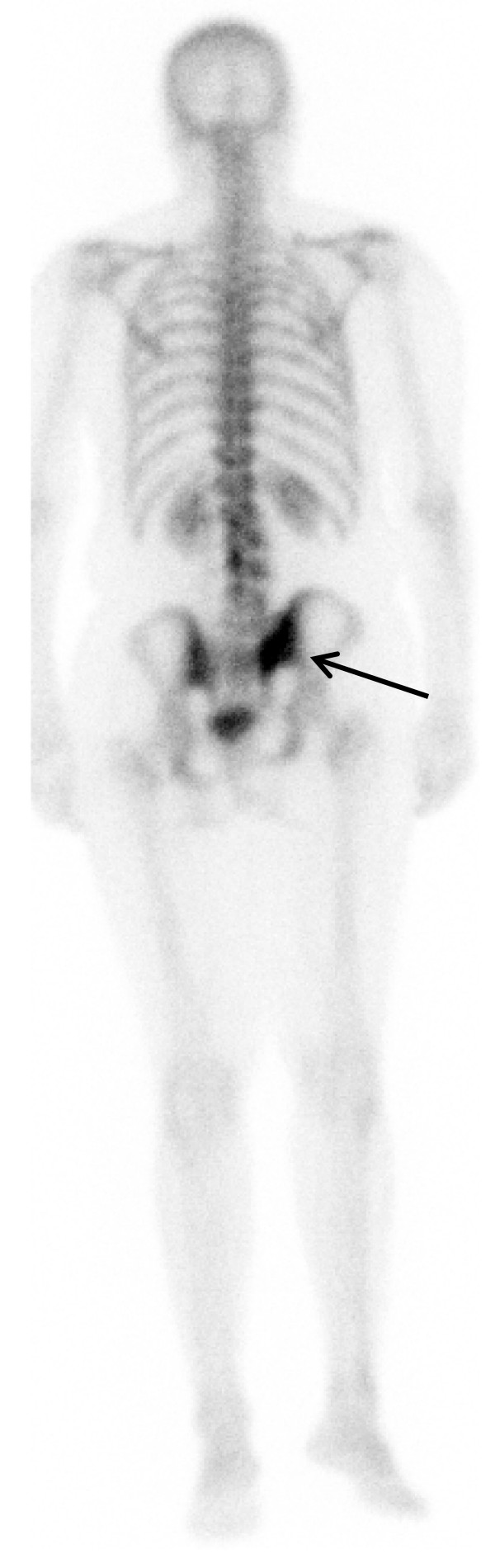
**Bone scan posterior view of a 54-year-old woman with a 2-year history of recurrent colon cancer revealed a focal tracer uptake in the right sacroiliac joint area suggesting possible bone metastasis**.

**Figure 2:**
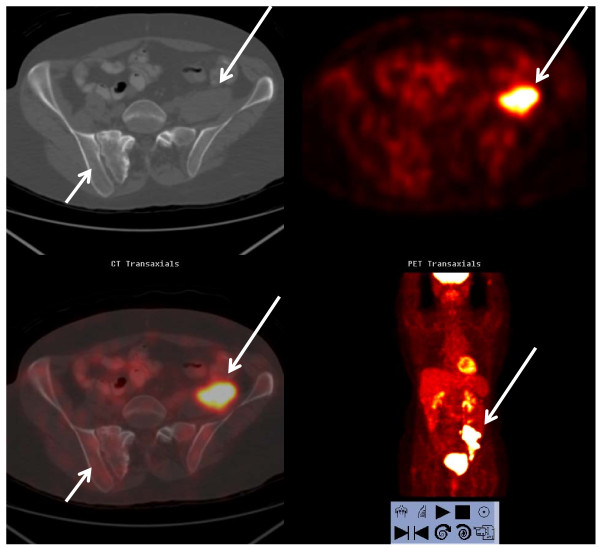
**FDG PET/CT scan of the same patient in Figure 1 revealed no focally increased FDG uptake or suspicious abnormalities to bone on the CT of the right sacroiliac area to correspond the bone scan findings (short arrows)**. The patient's history of pelvic fracture probably explained the focal tracer uptake in bone scan. The recurrent colon cancer in the left pelvis observed by both PET and CT scans is marked by the long arrows.

Our study results also confirm this unique behavior. No cancer metastasized to bone without first metastasizing to the liver or lung in our study. This finding contrasts with a previous study on bone metastasis in colorectal cancer patients by Kanthan et al [8] in which 1.1% of colorectal cancer population developed isolated metastasis. However, that study used bone scans and plain radiography to detect lesions, and both methods have low specificity that may overestimate the actual incidence of bone metastasis. This possibility was demonstrated during our data acquisition, when PET/CT disproved the findings of a positive bone scan in the pelvis, by demonstrating that there was no indication of bone metastasis on either PET or CT scan. Patient's remote history of pelvic fracture probably led to the false positive result of the bone scan (Figures 1 and 2). Another case that originally appeared as bone metastasis to the pelvis on a bone scan was identified as local/direct tumor invasion into the sacrum on the PET and CT scans (data not shown).

It is also likely that the higher sensitivity and specificity of PET and CT for detecting early metastasis in liver and lung compared with traditional radiography may account for the strong correlation among lesions to the liver, lung, and bone in our study. Possibly, concurrent involvement of other organs did exist but went undetected in the patients who had only bone lesions in the Kanthan et al study [8]. However, confirming whether the complete absence of true isolated bone metastasis is characteristic of colorectal cancer will require a larger study population.

This study sought to identify whether disease progression to the lungs could predict metastasis to bone. Although lung lesions are of particular interest as a forerunner of future bone metastasis as evidenced by the short time span from lung metastasis to bone involvement, the lesions in bone always appear after metastasis to liver, lung, or (in a large percentage) both. As the average 5-year survival rate of colon cancer patients with metastasis to bone continues to be 8.1% [9], and on average, 67% of those who developed bone involvement during this survey were dead 16 months after detection of bone metastasis, the importance of recognizing disease progression and potential significance of bone metastasis cannot be overemphasized.

### Disease Progression

The findings in this study regarding colon cancer metastasis to the bone and lungs correlate with results in the current literature. Our findings of lung metastasis in 25% of patients and bone metastasis in 5.5% of patients concur with the results from other studies [2,8]. As reported in the literature that colorectal cancer metastasizes first to the liver or lungs, which both contain dense capillary beds that can trap tumor cells and seed them into these organs [3]. The environment of a specific organ and its influence on tumor cell adhesion can also contribute to the efficacy of tumor spread, which occurs in colorectal cancer patients most frequently in the liver and lungs [2,3].

However, the 30% incidence of liver metastasis in our study is much lower than the incidence of liver metastasis in earlier studies, which reported liver involvement in 70–83% of patients [2,8]. The lower incidence of liver lesions detected in this study may be due to patient selection, because clinicians had resected the primary tumors in most of our patients and were monitoring them to evaluate possible recurrence during the investigation. During the study period, 44% of those who developed liver metastasis remained free of other organ metastasis, and 84% had liver lesions that never progressed to bone.

The percentage of metastatic lung involvement in our study correlates with that reported in the literature. However, unlike people with liver lesions, 78% of people with lung lesions showed concurrent disease involvement in other organs. When combined with the temporal difference between the time from liver metastasis to bone metastasis and the time from lung metastasis to bone metastasis, this finding suggests that lung metastasis indicates refractory, potentially serious disease more precisely than liver metastasis does. Most lung and liver metastases that we observed consisted of multiple lesions. However, solitary lesion occurred more often in the liver than that in the lung.

Although this study attempted to uncover the relationship between the pattern of disease progression and the development of bone metastasis, the resulting subpopulation of patients with metastasis to bone was small, and most had diffuse involvement of multiple organs at the same time point. Thus, further studies with a larger patient population with more frequent imaging studies are necessary to ascertain whether the order of organ involvement has any impact on the development of bone metastasis.

Lymphatic spread and local recurrence were excluded from analysis in this study because most patients had undergone local resection with local lymph node dissection before their staging work-up. Further investigation of the possible relationship between the nodal spread of disease, including local or distant nodal metastasis and metastasis to bone in a different patient population setting, would be interesting. After cancer cells break through the defense barrier of the lymphatic system, especially those lymph nodes outside the portal system, the cancer may metastasize more readily to the lungs and bone. We did not analyze brain metastasis because of the low sensitivity of PET for brain tumor detection and our PET/CT imaging protocol does not include the brain.

### Strengths

A strength of this study is that it included a systematic approach to evaluate disease progression. Using the files of 3 nuclear medicine radiologists facilitated the creation of a large database that spanned institutions, widened demographics, and maintained quality and consistency in the imaging reports.

In addition, unlike most studies that have characterized colorectal cancer metastasis, we used PET and CT, which are newer and more sensitive tools to detect colon cancer metastasis, rather than less precise methods like traditional radiography or bone scintigraphy [8]. Our results are therefore a direct reflection of what diagnosticians can expect to see when staging a colorectal cancer patient using current technology.

### Drawbacks

A major limitation of this study is the retrospective nature. We obtained the imaging studies during staging and subsequent follow-up by using a combination of PET with correlation of a recent CT and PET/CT. In addition, the imaging modality at each time point varied among patients and we only followed the patients during the imaging periods because of the retrospective nature of the study.

Most of the patients from the 3 outlying medical facilities experienced surgical resection of the primary tumor along with removal of neighboring lymph nodes before they had a detailed staging work-up. This patient population therefore may not be representative of general colorectal patients.

## Conclusion

The main implication of this study for clinicians is that if a colorectal cancer patient has no sign of disease involvement in the liver or lungs, evaluation of a suspicious bone lesion should not be aggressive, but should obtain more noninvasive data from other diagnostic modalities or follow-up.

Lung metastasis indicates the potential for cancer to metastasize to bone in the colorectal cancer population better than liver metastasis does.

## Competing interests

The authors declare that they have no competing interests.

## Authors' contributions

DQW designed the concept of this study and participated in manuscript writing. ESR participated in its design, collected the data and drafted the manuscript. DTZ participated in the study design, collected the data, and revised the manuscript. BJB, UAJ, and IWG provided the study material, gave administrative support, and revised the manuscript. All authors read and approved the final manuscript.

## Pre-publication history

The pre-publication history for this paper can be accessed here:

http://www.biomedcentral.com/1471-2407/9/274/prepub
